# Environmental variation mediates the prevalence and co-occurrence of parasites in the common lizard, *Zootoca vivipara*

**DOI:** 10.1186/s12898-019-0259-3

**Published:** 2019-10-22

**Authors:** Qiang Wu, Murielle Richard, Alexis Rutschmann, Donald B. Miles, Jean Clobert

**Affiliations:** 1 CNRS, Station d’Ecologie Théorique et Expérimentale, UMR 5321 and Université Toulouse III-Paul Sabatier, 09200 Moulis, France; 2 Université Fédérale Toulouse Midi-Pyrénées, 31013 Toulouse, France; 3 School of Biological Sciences, University of Auckland, Auckland, New Zealand; 4 Department of Biological Sciences, Ohio University, 131 Life Sciences Building, Athens, OH 45701 USA

**Keywords:** Parasites, Co-occurrence, Competition, Environmental mediation, Common lizard

## Abstract

**Background:**

Hosts and their parasites are under reciprocal selection, leading to coevolution. However, parasites depend not only on a host, but also on the host’s environment. In addition, a single host species is rarely infested by a single species of parasite and often supports multiple species (i.e., multi-infestation). Although the arms race between a parasite and its host has been well studied, few data are available on how environmental conditions may influence the process leading to multiple infestations. In this study, we examine whether: (1) environmental factors including altitude, temperature, vegetation cover, human disturbance, and grazing by livestock affect the prevalence of two types of ectoparasites, mites and ticks, on their host (the common lizard, *Zootoca vivipara*) and (2) competition is evident between mites and ticks.

**Results:**

We found the probability of mite infestation increased with altitude and vegetation cover, but decreased with human disturbance and presence of livestock. In contrast, the probability of tick infestation was inversely associated with the same factors. Individuals with low body condition and males had higher mite loads. However, this pattern was not evident for tick loads. The results from a structural equation model revealed that mites and ticks indirectly and negatively affected each other’s infestation probability through an interaction involving the environmental context. We detected a direct negative association between mites and ticks only when considering estimates of parasite load. This suggests that both mites and ticks could attach to the same host, but once they start to accumulate, only one of them takes advantage.

**Conclusion:**

The environment of hosts has a strong effect on infestation probabilities and parasite loads of mites and ticks. Autecological differences between mites and ticks, as indicated by their opposing patterns along environmental gradients, may explain the pattern of weak contemporary interspecific competition. Our findings emphasize the importance of including environmental factors and the natural history of each parasite species in studies of host–parasite coevolution.

## Background

A parasite lives in or on the host, feeds on the host, shows some degree of adaptation to the host and usually does not cause immediate death of the host [1]. Parasites can alter various attributes of their hosts including behavior, physiology, and life history as well as modify patterns of sexual selection and population dynamics [2, 3], which in turn may influence host–parasite coevolution [4]. On the other hand, hosts may evolve defense strategies to mitigate the negative effects of parasites, including parasite avoidance behavior, immunity, resistance, and tolerance [5–7]. However, host defenses are not the only barrier to parasitic exploitation; multiple infections by different parasite species can also affect host–parasite dynamics [8, 9]. Since a host can be considered a finite resource for parasites [10], one can predict that either intra- or inter-specific competition occurs among parasites sharing the same host species. For example, in a manipulative study, one flea species exhibited reduced developmental success in the presence of a competing species during a food shortage [11]. In addition to food resources, studies of ‘crowding effect’, i.e. the size of parasites is inversely proportional to the number of parasites in a given infection, suggest that alternative limiting factors may also affect competitive interactions between parasites. Such factors may include oxygen or space, as observed in a study on tapeworms [12]. The majority of evidence supporting the patterns of interspecific competition among parasites is based on endoparasite species [11], yet there is limited evidence of competition among ectoparasites. Therefore, it is of interest to investigate interspecific competition between ectoparasites and potential mediating factors (we hereafter use the term ‘infestation’ instead of ‘infection’, as ‘infestation’ conveys the idea of external attachment which is more appropriate for ectoparasites).

Ectoparasitic infestation is affected by both biotic attributes and characteristics of their local environment. Temperature and humidity may influence parasite activity and reproductive success [13, 14]. Altitude, vegetation structure, and topography may alter encounter rates between hosts and parasites [13–16]. Finally, anthropogenic disturbance from climate warming [17], habitat fragmentation [18], and habitat degradation [19] may also affect host–parasite dynamics. To date, several factors mediated by anthropogenic disturbance are known to influence host–parasite relationships [17, 20]. For example, habitat degradation from land use practices (e.g., livestock grazing) can reduce an individual’s ability to resist infestation through the decline in habitat quality, food availability, and refugia [21]. Livestock can also enhance local prevalence of parasites, because of their role in supporting survival and reproduction of some parasites [20, 21]. In addition, degradation of the thermal environment induced by climate change and habitat modification [17, 22] can also alter the microhabitat experienced by parasites in the off-host environment. Such environmental alternations have a cascading influence on host–parasite interactions, which can include broadening the distributional range of parasites, increasing the duration of activity of parasites, and enhancing host susceptibility through reductions in host condition [17, 23, 24]. Therefore, both natural environment and human-induced environmental variation need to be considered when analyzing the effects of parasites on host population dynamics.

Many reptile species are susceptible to infestations by ectoparasites, and mites and ticks are two of the most common ectoparasites of lizards [25]. In particular, common lizards (*Zootoca vivipara*) are often infested by mites of the genus *Ophionyssus* and sheep ticks *Ixodes ricinus*. Preliminary data showed that lizards exhibited the simultaneous presence of both mites and ticks at our study sites (personal observation). Thus, there is the potential for interspecific competition between these two types of ectoparasites.

Previous studies have examined the consequences of ectoparasitism on common lizards, such as the effect of host density on host–parasite interactions [26], the effect of maternal parasite load on offspring life-history traits [27], and the impact of maternal infestation on offspring performance and dispersal [28]. However, whether different environments may influence the prevalence of mites and ticks and their competitive interactions remains unknown in this species. The aim of this study is to investigate the organismal and environmental factors associated with parasite prevalence and the potential for competition between mites and ticks. We collected data on body size, body mass, and parasite infestation on common lizards from twelve populations along an altitudinal and human-induced perturbation gradient.

## Results

### Influence of the environment on parasite infestation

We captured a total of 775 lizards. Of these, 167 (21.5%) individuals were infested by mites and 122 (15.7%) individuals were infested by ticks, and 31 individuals (4%) had both mites and ticks. Mite and tick prevalence also varied according to the host sex and capture year (Table 1). Mite prevalence was higher in 2014 for males and females than the other years, whereas 2012 had lower prevalence of ticks than the latter 2 years.

**Table 1:** Variation in the prevalence of mites and ticks among years

Parasite	Year	Male		Female		Overall	
		Number	%	Number	%	Number	%
Mite	2012	121	18.2	165	8.5	286	12.6
	2014	88	52.3	178	29.2	266	36.8
	2015	53	18.9	170	13.5	223	14.8
Tick	2012	121	11.6	165	10.3	286	10.8
	2014	88	21.6	178	14.6	266	16.9
	2015	53	26.4	170	18.8	223	20.6

Number is the total number of individuals examined per sex (for a grand total of 262 males and 513 females) and per year. The prevalence is given as the percent of sampled individuals with either mites or ticks

Based on the broken-stick criterion, only the first PC axis was retained (PC1) for further analyses. PC1 axis accounted for 63% of the total variation (Table 2). The loadings show that PC1 increased with altitude and vegetation cover, but decreased with human disturbance and grazing condition. We therefore considered positive PC1 scores to arrange sites with high vegetation and low disturbance, but negative PC1 scores represented sites with high levels of anthropogenic disturbance.

**Table 2:** Summary of the PCA based on environmental variables

	Axis 1
Loadings	
Altitude	0.82
Vegetation cover	0.81
Human disturbance	− 0.87
Grazing condition	− 0.67
Eigenvalue	2.52
Proportion variation	0.63

Loadings (correlations between the original variables and the eigenvectors), eigenvalues, and proportion of variation explained by the first principal component axis

To predict the infestation probability of mites and mite load, we retained 6 and 20 models, respectively (Additional file 1: Tables S1, S2). Similarly, we kept 12 and 24 models for the infestation probability of ticks and tick load, respectively (Additional file 1: Tables S3, S4). We used the model averaging procedure to determine the variables influencing the probability of parasite infestation and parasite load.

**Fig. 1:**
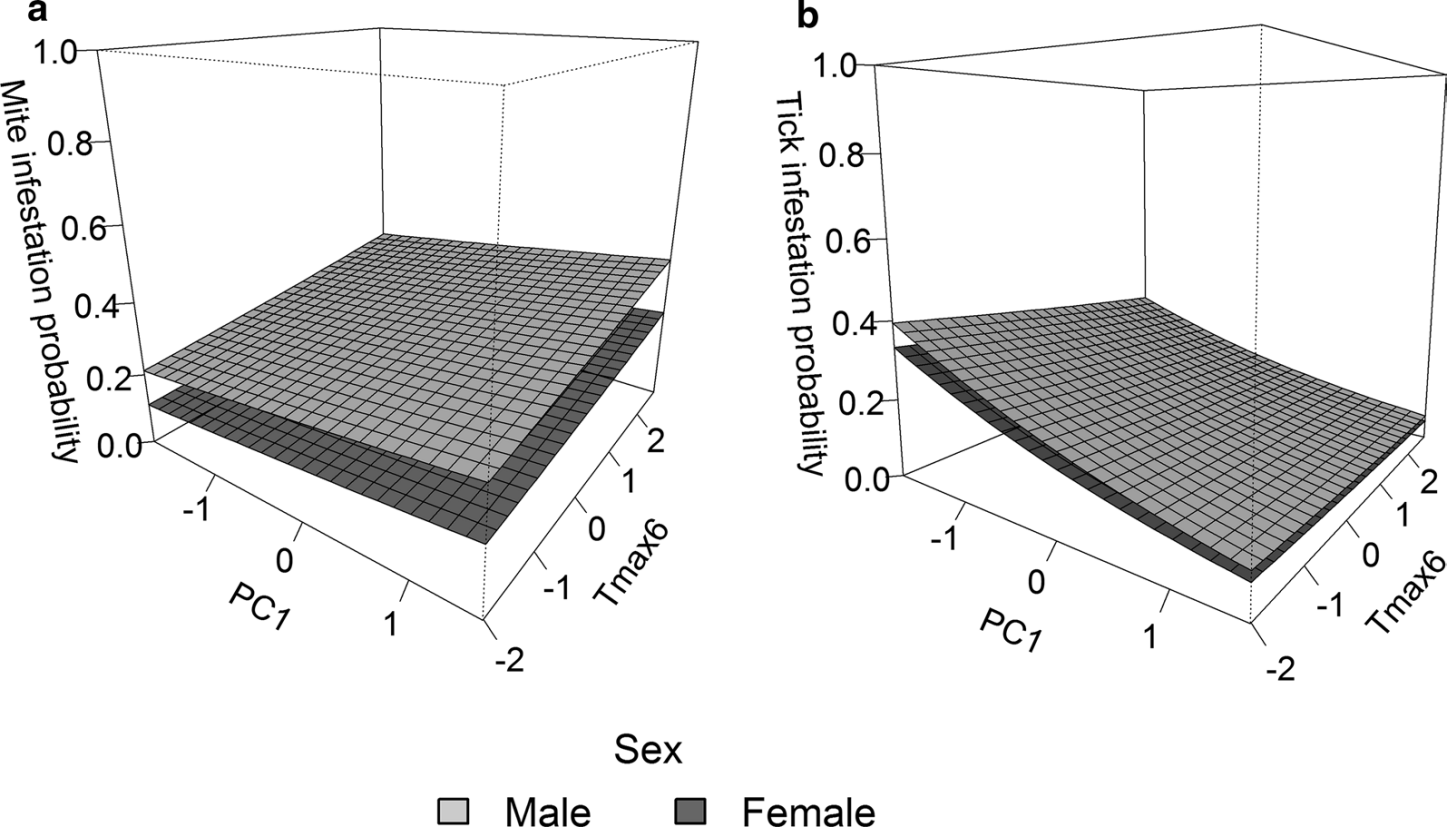
Infestation probability of parasites in relation to environmental factors according to host sex. **a** Estimates of probability of mite infestation in relation to PC1 and T_max6_; **b** estimates of probability of tick infestation in relation to PC1 and T_max6_. The infestation probability was the average value of three capture years

**Fig. 2:**
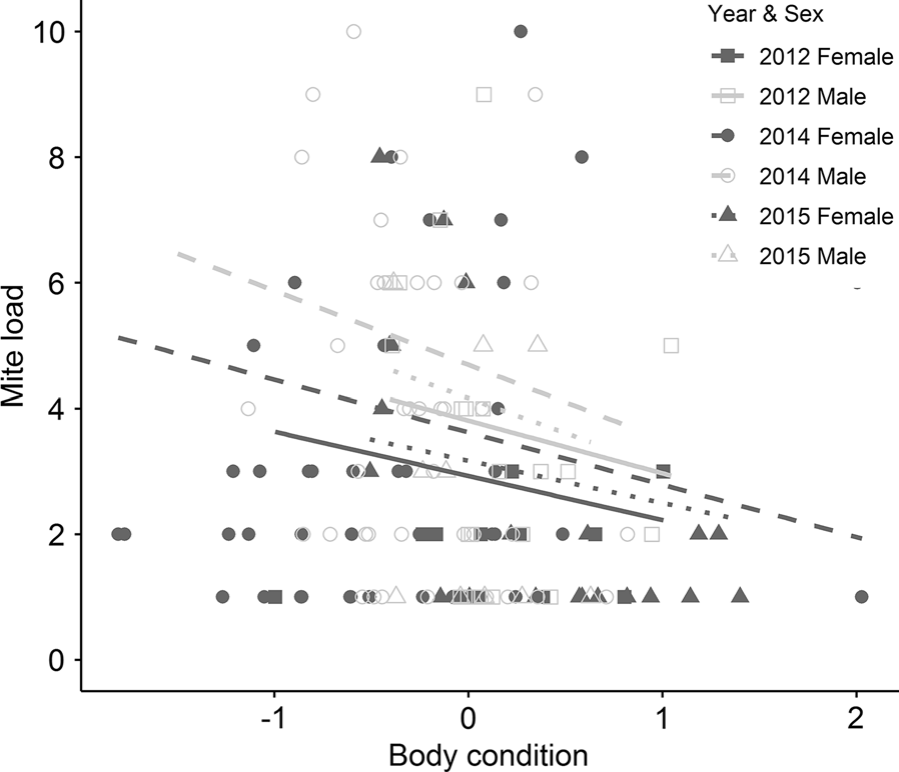
Mite parasite load in relation to host body condition by year and host sex

The probability of mite infestation was positively correlated with PC1 and male sex (Table 3, Fig. 1). Thus, individuals inhabiting sites with high altitude and high amounts of vegetation cover had a greater likelihood of mite infestation. In addition, males had a higher probability of mite infestation than females. In contrast, mite load was higher in males and individuals with lower body condition (Table 3, Fig. 2). Infestation probability and parasite load of mites were also associated with year: lizards captured in year 2015 had a higher infestation probability and higher parasite loads than in 2012 (Table 3).

**Table 3:** Relative importance and estimates for parameters predicting infestation probability and parasite load

	Infestation probability			Parasite load		
	IM	COEF	SE	IM	COEF	SE
Mites						
PC1	0.85	0.21*	0.10	0.32	0.06	0.04
T_max6_	0.62	0.17	0.10	0.23	0.01	0.04
Sex-m	1.00	0.81***	0.19	0.84	0.33***	0.08
Body condition	0.25	0.08	0.15	0.78	− 0.29***	0.08
Year 2014	1.00	1.58***	0.23	0.51	0.44**	0.13
Year 2015	1.00	0.41	0.27	0.51	0.22	0.15
Ticks						
PC1	1.00	− 0.52***	0.11	0.31	− 0.05	0.09
T_max6_	0.68	− 0.17	0.09	0.31	− 0.04	0.06
Sex-m	0.69	0.39	0.22	0.25	0.07	0.12
Body condition	0.27	0.06	0.15	0.22	− 0.03	0.11
Year 2014	0.83	0.51	0.26	0.55	0.02	0.14
Year 2015	0.83	0.64*	0.26	0.55	− 0.33*	0.16

Upper panel: infestation probability and parasite load for mites; Lower panel: infestation probability and parasite load for ticks

*IM* relative importance, *COEF* coefficients, *SE* standard error, *Sex-m* male sex, *T*_*max6*_ mean maximal temperature in June

Significant levels: * *P *< 0.05, ** *P *< 0.01, *** *P *< 0.001

Our analyses show that environmental factors explained the variation in tick infestation. The infestation probability of ticks was negatively correlated with PC1 (Table 3, Fig. 1), which suggests that lizards inhabiting grazed sites with higher human disturbance had a higher probability to be infested by ticks. Variation in tick loads was only related to year: lizards captured in 2015 had lower tick loads than in 2012 (Table 3).

### Co-occurrence of mites and ticks

**Fig. 3:**
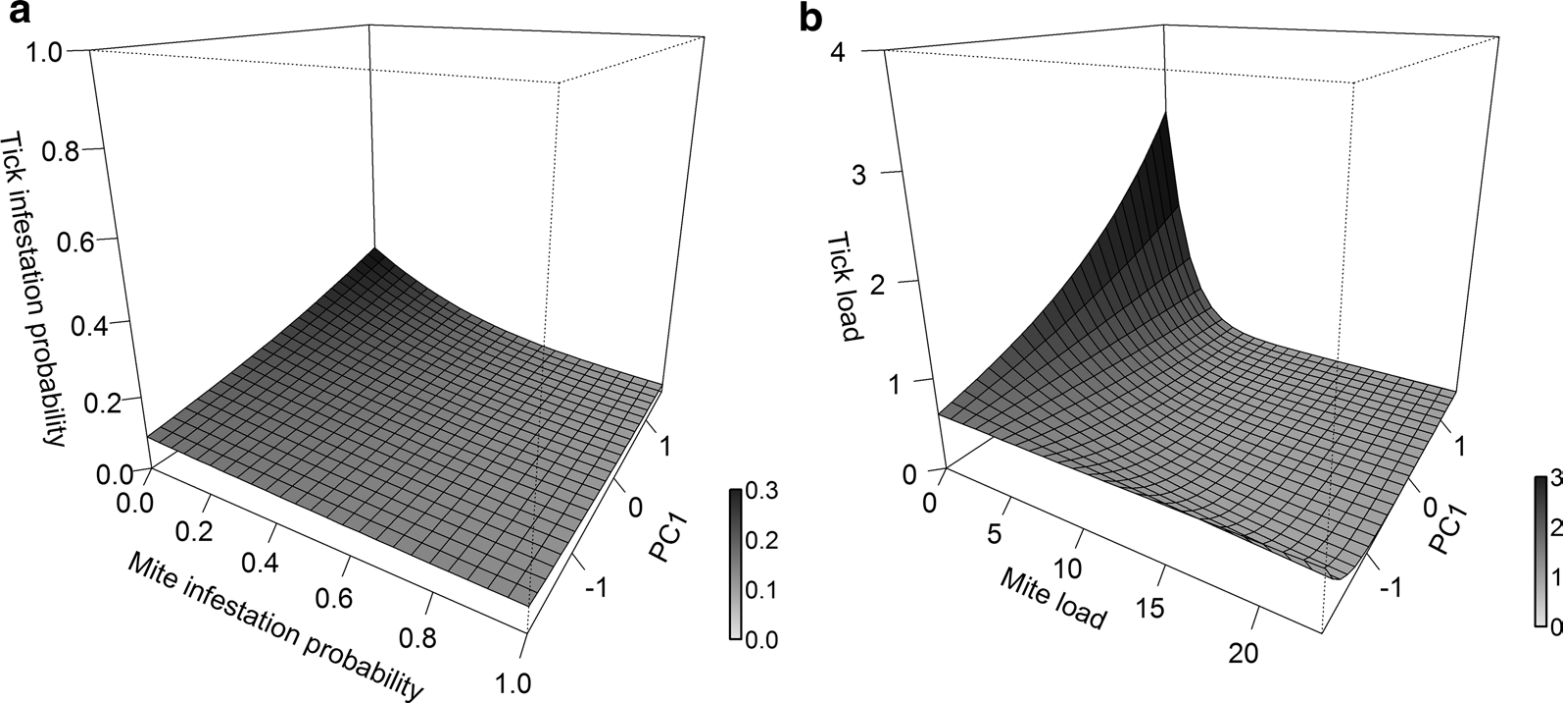
The relationship between mites and ticks under the mediation of environmental factors. **a** The probability tick infestation in relation to the probability of mite infestation and PC1; **b** parasite load of ticks in relation to parasite load of mites and PC1

We retained 7 and 2 models to estimate relationships between two types of parasites for either their infestation probability or parasite load, respectively (Additional file 1: Tables S5, S6). The probability of tick infestation was negatively associated with an interaction between mite infestation probability and PC1 (mite infestation probability × PC1, estimate = − 0.89 ± 0.31, *z *= − 2.90, *P* = 0.004, Fig. 3a). This result implies that mites can have a negative influence on ticks only through their interaction with the environment (PC1). In contrast, tick load, decreased with the interaction between mite load and PC1 (mite load × PC1: estimate = − 0.17 ± 0.04, *z *= − 3.70, *P* < 0.001, Fig. 3b). This result provides evidence that mite load had a direct, negative influence on tick load, and this relationship was mediated by the characteristics of the environment.

### Environments and competition between mites and ticks

**Fig. 4:**
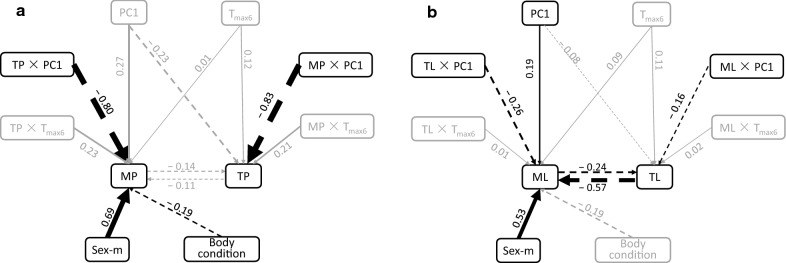
Structural equation model for effects of environmental factors and host traits on competition between parasites. **a** Probability of parasite infestation; **b** parasite load. Non- significant relationships (*P *> 0.05) are in grey, and black arrows indicate significant relationships (*P *< 0.05), solid when positive and dashed when negative. The thickness of the arrows indicates the strength of the relationship. Path coefficients are shown adjacent to the arrows (continuous variables were *Z*-transformed to obtain the standardized coefficients). *MP* probability of mite infestation, *TP* probability of tick infestation, *ML* mite load, *TL* tick load, *Sex-m* male sex, *PC1* the first PCA axis based on environmental factors

The piecewise SEM models for examining the probability of parasite infestation (Fisher’s *C* = 3.97, df = 4, *P* = 0.41) and parasite load (Fisher’s *C* = 2.76, df = 4, *P* = 0.60) exhibited high goodness of fit values. When examining the probability of infestation, there was no evidence of competition between mites and ticks. However, we found that the probability of infestation by mites was negatively associated with the interaction between ticks and PC1 and similarly, ticks were negatively related to the interaction between mites and PC1 (Fig. 4a). Hence, when considering the probability of infestation, mites and ticks appear to have a mutual and negative effect on one another, but the interaction is mediated through variation in environmental characteristics. When we focused on parasite load, we detected direct negative relationships between mite load and tick load (Fig. 4b). The SEM also confirms that the probability of mite infestation and mite load were influenced by the sex and body condition of hosts (Fig. 4b). The results from the SEM indicate that environmental factors mediated the probability of infestation (or parasite load) of two parasites in opposite directions, and the competition between mites and ticks depended on their relative parasite load.

## Discussion

In this study, we investigated the variation in the probability of infestation and parasite load of two ectoparasites of the common lizard. We measured the patterns of parasitism from 12 different populations inhabiting sites that differed in several environmental factors. Our analyses show that environmental factors affected the probability of infestation by mites and ticks. However, these two ectoparasites exhibited divergent responses to prevailing environmental conditions among the sample sites. The probability of mite infestation was positively correlated with PC1, which suggests that populations at high altitude and high vegetation cover had a higher chance of being infested by mites. In contrast, tick infestation probability was negatively correlated with PC1, which suggests that populations in open habitats with high disturbance and grazing condition were more susceptible to ticks. Mite load was higher in males and in individuals with low body condition, but no such pattern was obtained for ticks.

### Influence of the environment on parasite infestation

Our results reflect the differences in the autecology of each ectoparasite. The probability of mite infestation increased in sites at high elevations with greater vegetation coverage, but lower anthropogenic disturbance. Spoecker and Zippel et al. [15, 29] showed that the pattern of an increase in mite prevalence with elevation might result from the lower temperatures and mesic characteristics of high altitude habitats, rather than the elevation per se. Thus, altitude might be a factor which encapsulates multiple elements, such as temperature, precipitation, relative humidity, and vegetation cover. Our results contradict this pattern, because we found no association between altitude and mean annual precipitation in study sites (*r* = − 0.14, *P *= 0.72). However, the lack of a correlation is not unexpected, because the sites were selected for studying the effect of temperature and anthropogenic disturbance while minimizing the variation in other variables such as humidity.

Our analyses also reveal that higher plant cover correlated with a higher probability of infestation by mites. Parasite prevalence is often associated with habitat characteristics, such as vegetation structure [30]. Dense vegetation may provide sheltered microhabitats for parasites, therefore increasing local density, which can then result in higher rates of infestation of lizards [31]. In addition, these are the same microhabitats exploited by common lizards. Microclimate data will be needed to further investigate this hypothesis. However, the negative correlations between altitude and body condition (*r *= − 0.09, *P* = 0.015) and between vegetation cover and body condition (*r *= − 0.13, *P* = 0.0004) may provide other explanations. Given that mite infestations are greater in lizards with lower body condition (Table 3), the observed elevational and vegetational patterns may be a consequence of lizards with lower body condition inhabiting sites at higher altitude and great vegetation cover.

Patterns of tick infestation have been shown to be sensitive to the presence of large herbivores in grazed pastures. Adult female ticks require large animals as a host for their survival, reproduction, and maintenance of populations [24]. Therefore, the pattern of lizards having higher rates of infestation in grazed habitats might be explained by the enhanced survival and reproductive success of ticks in these areas. Hence, the abundance of larvae and nymphs available to feed on lizards should also be higher [32]. Another non-exclusive explanation is human disturbance per se. Increased anthropogenic disturbance such as human activity or tourism can more frequently stimulate the anti-predatory behavior of lizards and in turn reduce the energy allocated to their body condition and immune response [19]. We did not find support for this hypothesis since body condition was only weakly associated with human disturbance (*r *= 0.06, *P* = 0.12). However, a subsequent experiment revealed that anti-predatory behaviors of lizards exhibit a concomitant change with anthropogenic perturbations in our study sites (Qiang et al. unpublished data). It still remains to be investigated whether such behavioral changes result in a reduction of body condition and lowered immunity.

Finally, with the exception of five environmental factors examined above, the probability of mite and tick infestation varied among years in our populations. This suggests that other unmeasured abiotic or biotic factors also affect parasite prevalence. For example, humidity (including precipitation levels) [33], seasonality [34], and host population dynamics [35] can affect parasite prevalence in other species.

### Parasite infestation and host characteristics

We found that the probability of mite infestation and mite load were higher in males than females. Male-biased infestation is common in many animal species and various explanations have been proposed in the literature [36]. For example, larger home ranges and increased mobility of males during the reproductive season is likely to raise the probability of encountering parasites [37, 38]. In addition, many other life history traits such as lifespan, mating system, behavior, social structure, immunity, and sex steroids are all potential explanations of male-biased infestations observed in our study [36, 39–41]. Androgens are known to affect parasite prevalence. For instance, high testosterone levels in males enhance their mating success, but is accompanied by a concomitant suppression of immune function, which may induce a higher infestation by parasites (i.e. the immune-competence handicap hypothesis) [39]. Testosterone also elicits more aggressive behaviors in individuals and results in a simultaneous higher cost of energy or even mortality, which might explain the higher susceptibility of males to parasite infestation [37]. The pattern of testosterone induced behavioral changes has partial supports in our study system as males are usually more aggressive than and dominant over females in common lizards [42]. However, there are also counter-examples on the role of testosterone, such as female-biased parasitism [43] and even opposite effects of testosterone on different types of parasites [44]. More detailed studies on the causal relationship between testosterone, aggressive behavior, and parasitism are therefore needed in this species to better understand differences between males and females in parasitism.

Irrespective of their sex, lizards with lower body condition were more susceptible to a higher mite load. In common lizards, individuals with poor body condition [45] usually have lower metabolic rates and may fail to express a strong immune response [46], and hence a higher accumulation of parasites. It may also be possible that parasites cause reductions in body condition of lizards, which in turn induces a higher infestations rate. Further manipulative experiments are needed to disentangle the underlying cause–effect relationship between host body condition and parasitism.

### Competition between mites and ticks

Out of the 775 lizards examined, we found a small percentage (~ 4%) of individuals with a co-infestation of mites and ticks. This pattern reflects the differences in environmental factors associated with the life cycles of mites and ticks. We hypothesize that the difference in the natural history of these two ectoparasites decreases the opportunity for competition. Competition between mites and ticks might further be avoided as a result of microhabitat selection on the host: ticks mainly attach on the neck and around the forelimbs of lizards [38, 47], whereas mites occur on the ventral scales of lizards [48]. This explanation is consistent with our observations in common lizards. However, even if competition for habitat or space is unlikely, there might be competition for resources as both types of parasites feed on the blood of lizards. Our results point out a negative association for parasite load but not for infestation probability. This suggests that both types of parasites may infest the same host but once the host is in low body condition, only one type parasite seems to take advantage. However, the negative correlation of parasite load between the two types of parasites, although significant, was weak, and so this result should be interpreted with caution. Additionally, variation in the probability of tick infestation seems to be a consequence of indirect effects of the interplay between mites and environment, rather than a direct suppressive effect of mites (Fig. 4). This result confirms that the relationship between the two parasites was mediated by environmental factors, which attenuated the opportunity for competition. We suggest that when each parasite is regulated by distinct habitat constraints then competition among parasites has a rather weak effect on overall prevalence and parasite load.

## Conclusion

We demonstrated that the probability of infestation by mites and ticks was mediated by divergent environmental factors. The distinct natural histories of these parasite species might explain why we found limited evidence for competition between them. The probability of infestation and parasite load of mites were influenced by the host sex and host body condition. Our results highlight the importance of how environmental variation and the autecology can mediate, to a large extent, the interaction between different types of parasites. We emphasize that further experimental manipulation should provide better inferences about the existence of competition between parasites.

## Methods

### Study system

The common lizard (*Zootoca vivipara*) is a small, viviparous lizard (adult snout-vent length varies from 40 to 60 mm in males and 45 to 75 mm in females) with a broad geographic distribution extending throughout Europe and Asia [49]. It inhabits mesic habitats, such as peat bogs, meadows, and heathlands. Males emerge from hibernation prior to females in late April–early May. Mating starts in early May following the emergence of females. Parturition occurs two and a half months later [27].

Our focal parasite speccies include mites of the genus *Ophionyssus* [26] and sheep ticks *Ixodes ricinus.* These two haematophagous arachnids have a free living stage in the soil and attach to a host for a blood meal [25]. They also have similar life cycles including larval, nymph, and adult stages [50, 51]. *Ixodes* ticks are sensitive to desiccation and their preferred habitats relate to multiple factors including vegetation cover, climate, and availability of hosts. In contrast, *Ophionyssus* mites are both thermophilic and hygrophilic [50, 52]. The main hosts of ticks are large mammals such as deer, cattle, and sheep [24], whereas mites specialize on reptiles. Both ectoparasites are vectors for blood parasites [25, 53]. In our study sites, *Z. vivipara* is the main reptile host of these two parasites. Other potential, alternative reptile hosts, e.g., sand lizards (*Lacerta agilis*) and snakes are found at low densities in our study sites.

### Lizard sampling, morphometrics and parasite collection

**Fig. 5:**
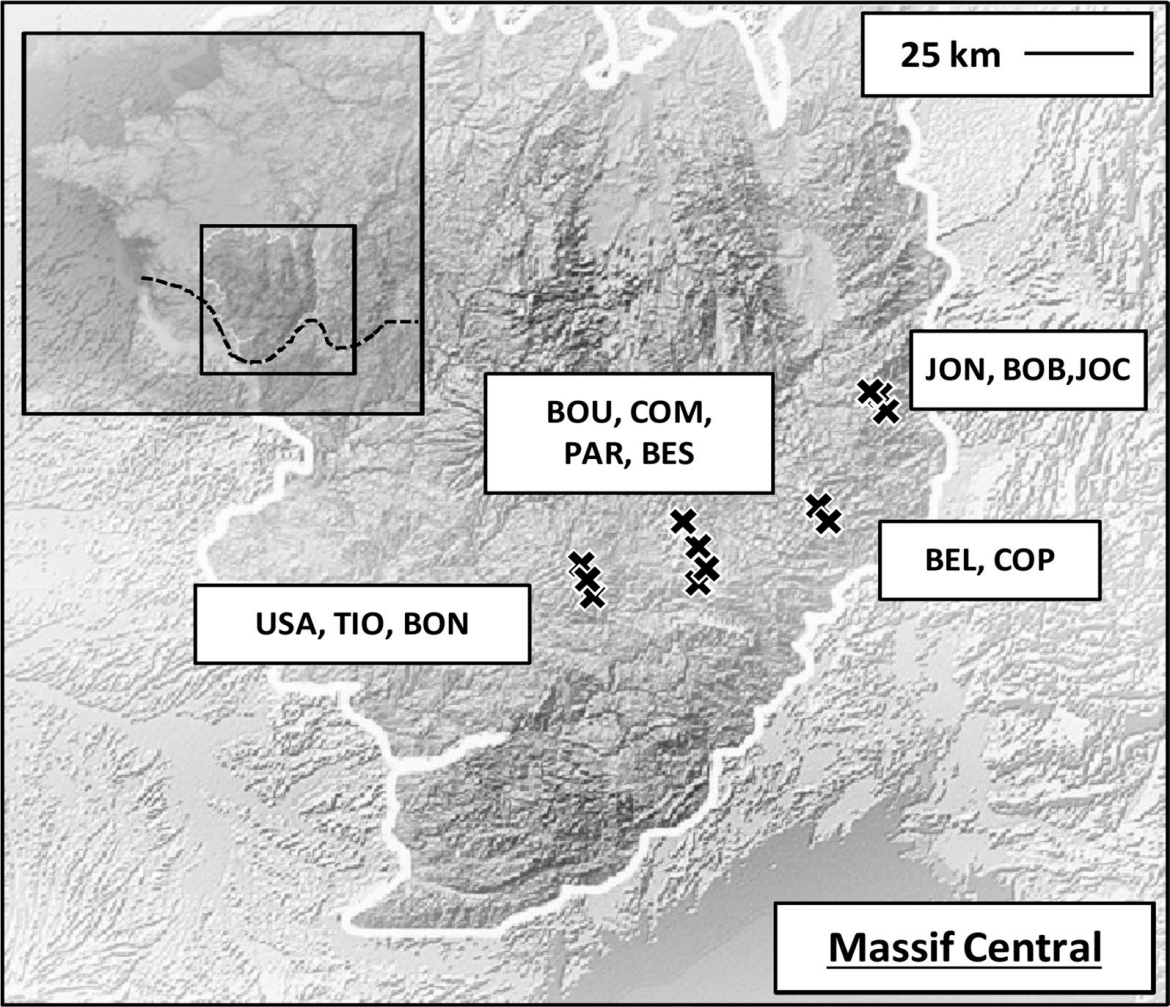
Location of the sample sites in the Massif Central, France. The white line delineates the Massif Central. The dashed line represents the southern boundary of the distribution of viviparous common lizards (this map was modified from [54])

We sampled lizards from twelve different sites (Fig. 5) in the Massif Central, France, in 2012, 2014, and 2015 (Table 4). The average date of capture was 26 June (± 5 days). At each locality, between 15 and 25 females and 10 males were captured by hand and transported to a field laboratory. We measured body size (Snout-to-vent length, SVL ± 0.5 mm) and body mass (± 0.1 g) of each lizard. We estimated body condition separately for each sex as the residuals from a linear regression of body mass versus SVL (males: intercept = − 3.31 ± 0.35, slope = 0.13 ± 0.006, *t *= 19.63, *P *< 0.001, adjusted R^2^ = 0.60; females: intercept = − 5.91 ± 0.40, slope = 0.17 ± 0.006, *t *= 26.64, *P *< 0.001, adjusted R^2^ = 0.58). Following capture, each lizard was visually examined for the presence of ectoparasites and numbers of mites and ticks were recorded. Mites always occurred under the ventral scales, whereas ticks tend to attach on the area behind the ear openings to the base of the neck and the forelimbs.

**Table 4:** Description of the study sites with related mountain range and environmental factors

Site	Mountain range	SB	N	Alt (m)	T_max6_ (°C)	VCI	HD	G
BOB	Mont du Vivarais	2	68	1450	21.02	0.04	2	0
JOC	Mont du Vivarais	1	40	1300	26.77	0.02	2	1
JON	Mont du Vivarais	2	91	1405	22.86	0.19	2	0
BEL	Mont du Velay	2	55	1350	18.82	0.30	1	0
COP	Mont du Velay	3	86	1360	21.95	0.07	2	0
BON	Mont d’Aubrac	1	34	1340	17.18	0.02	2	1
TIO	Mont d’Aubrac	2	75	1300	17.96	0.00	2	1
USA	Mont d’Aubrac	1	36	1210	15.85	0.05	3	1
BES	Margeride	2	64	1220	21.63	0.10	3	1
BOU	Margeride	2	72	1410	19.60	0.12	2	1
COM	Margeride	3	80	1435	20.04	0.19	1	1
PAR	Margeride	2	74	1415	20.57	0.32	1	0

Each site is described with number of sampling bouts (SB), sample size (number of individuals, N) for each population (for a total of 775 lizards) and environmental factors including altitude (Alt), mean maximal temperature in June (T_max6_), vegetation cover index (VCI), human disturbance (HD, 1 to 3, with 1 being the least disturbed), and grazing condition (G: 0 = ungrazed, 1 = grazed)

### Environmental correlates of parasite infestation

At each capture site, we recorded the altitude, the maximum temperature during June (T_max6_), an index of vegetation coverage, an index of anthropogenic disturbance, and the presence or absence of livestock (grazing). Altitude was the mean altitude of each site. The maximum temperature of June was based on measurements from the nearest meteorological station. However, as there was no unique meteorological station for each study site, we measured the local temperature of all sites by using temperature data loggers (Thermochron iButtons©, Waranet Solution, Auch, France, see Rutschmann et al. [55]). The final maximum June temperature in the analysis was predicted by the coefficients of a linear regression between temperature estimated by data loggers and those from the nearest meteorological station [55]. The vegetation cover index was derived from aerial photographs (scaled Google Earth© views, Mountain View, CA, USA; the images were accessed on 11th Jan 2015) and calculated as the proportion of pixels representing trees or shrubs within the total capture area [55]. Our index of habitat disturbance involved a rank order from 1 to 4, with 1 being the least disturbed site and 4 being the highest disturbed one. Grazing condition was represented by 0 or 1, with 0 indicating no grazing and 1 indicating the presence of livestock on the site (we do not consider other ungulates such as the roe deer, *Capreolus capreolus*, because these species are uncommon at our sites).

### Statistical analysis

#### Influence of the environment on parasite infestation

The variables we used to characterize the environment of each capture site have different scales of measurement. Thus, we used a principal components analysis (PCA) on four environmental factors (altitude, vegetation index, human disturbance and grazing condition) to generate new axes for describing differences among sites (Table 4, [55]). We extracted principal components from a correlation matrix using the function *principal* in the ‘psych’ package in R [56]. We determined the number of PC axes to retain based on the broken-stick method [57]. We used the PC axes to characterize the environmental features of each sample site.

We used the PC scores to investigate the relative roles of host phenotypic characteristics and environmental factors in structuring the susceptibility to parasitism on common lizards. Following the method of hurdle models [58], we used generalized linear models (GLM) to examine the probability of parasite infestation (Binomial distribution, uninfested vs. infested) and parasite load (zero-truncated Poisson distribution, the number of parasites found on an infested individual). We included the following predictor variables: the first PCA axis based on the environmental variables, T_max6_, year, sex, and body condition. We also performed a generalized linear mixed model (GLMM) analysis that used capture site as the random factor. When including all 3 years in the analysis, the GLMM model failed to converge. We attribute this to unbalanced sampling of some sites during 2015. We repeated the mixed model analysis, but excluded the data for these sites (110 lizards, accounting for 14% of total sample size), and the model yielded similar results to the GLM. When needed, we also included a scale parameter [59] to compensate for the overdispersion of mite and tick loads in the model selection procedure (ratio between residual deviance and residual degrees of freedom, *ĉ *= 3.55 and 1.29 respectively). We checked for the presence of multicollinearity among the variates by calculating the variance inflation factors (VIF) using the *vif* function in the car R package [60] following recommendations in Zuur et al. [61]. All variance inflation factors were below 3.0, which suggested no effects of multicollinearity.

We used the Akaike information criterion (AIC or to correct for overdispersion, QAIC) for model selection. When several models had similar AIC values, we conducted model averaging using the ‘MuMIn’ R package [62]. Two approaches to choose candidate models can be found in the literature: (1) when ∆AIC ≤ 2 or (2) cumulative Akaike model weights ≤ 95%. These two methods yielded similar results in our data, and we chose the latter one as it furnishes a more precise estimate of the support for each possible model (see more descriptions in [63]).

#### Co-occurrence of mites and ticks

We used generalized linear mixed models [64], with the infestation probability of ticks (or tick load) as the response variable and the infestation probability of mites (or mite load), the first environmental PC axis, T_max6_, and their interactions included as predictor variables. We added capture site as a random factor, and we also included an observational-level random factor in the parasite load model to account for the overdispersion. The selection of candidate models was based on their AIC values and with model averaging procedures aforementioned.

#### Environments and competition between mites and ticks

We used piecewise structural equation modeling (SEM) to explore the causal relationships between environmental factors, host traits, and potential competition between two parasites. We built models either for infestation probability or parasite load based on a priori observations and results, using the ‘piecewiseSEM’ package in R [65]. Compared to the traditional SEM, the piecewise SEM can account for the non-normal distribution of variables and random structure in models. Furthermore, the method allows a test of whether any paths are missing from the model by using Shipley’s test of d-separation [66]. The goodness-of-fit of piecewise SEM was also evaluated with the akaike information criterion (AIC). The adequacy of overall fit is indicated by a non-significant *P* value based on a Chi square test (*P *> 0.05) and AIC [67].

## Supplementary information

**Additional file 1.** Additional tables.

## Data Availability

The datasets used and analyzed during the current study are available from the corresponding author on reasonable request.

## Abbreviations

AIC: akaike information criterion
GLM: generalized linear model
GLMM: generalized linear mixed model
PCA: principle component analysis
PC1: first PCA axis
SEM: structural equation modeling
VIF: variance inflation factors
