# Supplementary material for: Environmental variation mediates the prevalence and co-occurrence of parasites in the common lizard, Zootoca vivipara
Source: BMC Ecol. 2019 Oct 22;19:44. doi: 10.1186/s12898-019-0259-3 (PMC6806499; doi:10.1186/s12898-019-0259-3)
Supplement: Supplementary file 1 — Additional file 1. Additional tables. [file 12898_2019_259_MOESM1_ESM.docx]

**Additional Tables**

**Table S1**. Candidate models for predicting the probability of mite infestation under the effects of environmental factors and host traits (A total of 6 models’ accumulative Akaike weight ≤ 0.95).

| Model | logLik | *k* | AIC | ΔAIC | *w_i_* | Intercept | Year | PC1 | T_max6_ | Sex-m | | Body condition |
| --- | --- | --- | --- | --- | --- | --- | --- | --- | --- | --- | --- | --- |
| 1 | -363.54 | 6 | 739.07 | 0.00 | 0.339 | -2.4108 | + | 0.2022 | 0.1727 | + | — | |
| 2 | -364.95 | 5 | 739.91 | 0.83 | 0.224 | -2.3797 | + | 0.2100 | — | + | — | |
| 3 | -363.37 | 7 | 740.75 | 1.67 | 0.147 | -2.4293 | + | 0.2098 | 0.1762 | + | 0.0845 | |
| 4 | -365.81 | 5 | 741.61 | 2.54 | 0.095 | -2.3780 | + | — | 0.1789 | + | — | |
| 5 | -364.85 | 6 | 741.70 | 2.62 | 0.091 | -2.3935 | + | 0.2160 | — | + | 0.0676 | |
| 6 | -367.48 | 4 | 742.96 | 3.89 | 0.049 | -2.3471 | + | — | — | + | — | |

Abbreviations: AIC = Akaike’s information criterion; logLik = Likelihood; *k* = Number of parameters in the model; *w_i_* = Model weight; PC1 = The first PCA axis to describe environmental factors; T_max6_ = Mean maximal temperature of June; Year = Capture year; Sex-m = Male sex.

**Table S2**. Candidate models for predicting mite load under the effects of environmental factors and host traits (A total of 20 models’ accumulative Akaike weight ≤ 0.95).

| Model | logLik | *k* | AICc | ΔAICc | *w_i_* | Intercept | Year | PC1 | T_max6_ | Sex-m | Body condition |
| --- | --- | --- | --- | --- | --- | --- | --- | --- | --- | --- | --- |
| 1 | -482.99 | 3 | 280.96 | 0.00 | 0.193 | 1.1603 | — | — | — | + | -0.3354 |
| 2 | -476.93 | 5 | 281.82 | 0.86 | 0.126 | 0.8889 | + | — | — | + | -0.2439 |
| 3 | -482.62 | 4 | 282.88 | 1.92 | 0.074 | 1.1583 | — | 0.0307 | — | + | -0.3200 |
| 4 | -482.95 | 4 | 283.07 | 2.10 | 0.068 | 0.8631 | + | — | — | + | — |
| 5 | -482.97 | 4 | 283.08 | 2.12 | 0.067 | 1.1615 | — | — | -0.0076 | + | -0.3350 |
| 6 | -475.76 | 6 | 283.34 | 2.37 | 0.059 | 0.8452 | + | 0.0579 | — | + | -0.2130 |
| 7 | -479.94 | 5 | 283.52 | 2.56 | 0.054 | 0.7988 | + | 0.0900 | — | + | — |
| 8 | -491.60 | 2 | 283.72 | 2.76 | 0.049 | 1.3273 | — | — | — | — | -0.3194 |
| 9 | -476.87 | 6 | 283.96 | 3.00 | 0.043 | 0.8839 | + | — | 0.0148 | + | -0.2421 |
| 10 | -486.19 | 4 | 284.89 | 3.93 | 0.027 | 1.1088 | + | — | — | — | -0.2289 |
| 11 | -482.60 | 5 | 285.02 | 4.06 | 0.025 | 1.1595 | — | 0.0308 | -0.0083 | + | -0.3194 |
| 12 | -482.73 | 5 | 285.10 | 4.13 | 0.024 | 0.8538 | + | — | 0.0263 | + | — |
| 13 | -490.97 | 3 | 285.47 | 4.50 | 0.020 | 1.3217 | — | 0.0399 | — | — | -0.3001 |
| 14 | -475.70 | 7 | 285.51 | 4.55 | 0.020 | 0.8402 | + | 0.0579 | 0.0145 | + | -0.2114 |
| 15 | -479.79 | 6 | 285.61 | 4.65 | 0.019 | 0.7910 | + | 0.0894 | 0.0225 | + | — |
| 16 | -491.46 | 3 | 285.74 | 4.78 | 0.018 | 1.3285 | — | — | -0.0206 | — | -0.3186 |
| 17 | -491.89 | 3 | 285.99 | 5.03 | 0.016 | 1.0799 | + | — | — | — | — |
| 18 | -496.05 | 2 | 286.24 | 5.27 | 0.014 | 1.2218 | — | — | — | + | — |
| 19 | -485.01 | 5 | 286.38 | 5.42 | 0.013 | 1.0640 | + | 0.0582 | — | — | -0.1996 |
| 20 | -488.98 | 4 | 286.47 | 5.50 | 0.012 | 1.0161 | + | 0.0891 | — | — | — |

Abbreviations: AIC = Akaike’s information criterion; logLik = Likelihood; *k* = Number of parameters in the model; *w_i_* = Model weight; PC1 = The first PCA axis to describe environmental factors; T_max6_ = Mean maximal temperature of June; Year = Capture year; Sex-m = Male sex.

**Table S3**. Candidate models for predicting the probability of tick infestation under the effects of environmental factors and host traits (A total of 7 models’ accumulative Akaike weight ≤ 0.95).

| Model | logLik | *k* | AIC | ΔAIC | *w_i_* | Intercept | Year | PC1 | T_max6_ | Sex-m | Body condition |
| --- | --- | --- | --- | --- | --- | --- | --- | --- | --- | --- | --- |
| 1 | -314.65 | 6 | 641.31 | 0.00 | 0.245 | -2.3175 | + | -0.5122 | -0.1606 | + | — |
| 2 | -316.23 | 5 | 642.46 | 1.15 | 0.138 | -2.3312 | + | -0.5492 | — | + | — |
| 3 | -316.41 | 5 | 642.81 | 1.50 | 0.116 | -2.1376 | + | -0.5032 | -0.1654 | — | — |
| 4 | -314.55 | 7 | 643.11 | 1.80 | 0.100 | -2.3298 | + | -0.5083 | -0.1586 | + | 0.0667 |
| 5 | -318.07 | 4 | 644.13 | 2.82 | 0.060 | -1.9096 | — | -0.5351 | -0.1814 | + | — |
| 6 | -318.08 | 4 | 644.15 | 2.84 | 0.059 | -2.1464 | + | -0.5413 | — | — | — |
| 7 | -316.09 | 6 | 644.18 | 2.87 | 0.058 | -2.3455 | + | -0.5440 | — | + | 0.0776 |
| 8 | -319.25 | 3 | 644.50 | 3.19 | 0.050 | -1.7918 | — | -0.5245 | -0.1832 | — | — |
| 9 | -316.31 | 6 | 644.63 | 3.32 | 0.047 | -2.1490 | + | -0.4995 | -0.1635 | — | 0.0625 |
| 10 | -319.92 | 3 | 645.84 | 4.53 | 0.025 | -1.9072 | — | -0.5809 | — | + | — |
| 11 | -317.95 | 5 | 645.90 | 4.59 | 0.025 | -2.1596 | + | -0.5363 | — | — | 0.0727 |
| 12 | -318.06 | 5 | 646.12 | 4.81 | 0.022 | -1.9097 | — | -0.5341 | -0.1808 | + | 0.0175 |

Abbreviations: AIC = Akaike’s information criterion; logLik = Likelihood; *k* = Number of parameters in the model; *w_i_* = Model weight; PC1 = The first PCA axis to describe environmental factors; T_max6_ = Mean maximal temperature of June; Year = Capture year; Sex-m = Male sex.

**Table S4**. Candidate models for predicting tick load under the effects of environmental factors and host traits (A total of 24 models’ accumulative Akaike weight ≤ 0.95).

| Model | logLik | *k* | AICc | ΔAICc | *w_i_* | Intercept | Year | PC1 | T_max6_ | Sex-m | Body condition |
| --- | --- | --- | --- | --- | --- | --- | --- | --- | --- | --- | --- |
| 1 | -224.54 | 3 | 356.15 | 0.00 | 0.170 | 0.9727 | + | — | — | — | — |
| 2 | -228.33 | 1 | 357.78 | 1.63 | 0.075 | 0.8659 | — | — | — | — | — |
| 3 | -224.46 | 4 | 358.20 | 2.04 | 0.061 | 0.9500 | + | — | — | + | — |
| 4 | -224.53 | 4 | 358.31 | 2.16 | 0.058 | 0.9810 | + | 0.0113 | — | — | — |
| 5 | -224.54 | 4 | 358.31 | 2.16 | 0.058 | 0.9728 | + | — | — | — | -0.0133 |
| 6 | -224.54 | 4 | 358.32 | 2.17 | 0.058 | 0.9686 | + | — | -0.0054 | — | — |
| 7 | -227.38 | 2 | 358.41 | 2.26 | 0.055 | 0.8217 | — | -0.0860 | — | — | — |
| 8 | -227.41 | 2 | 358.45 | 2.30 | 0.054 | 0.8459 | — | — | -0.0640 | — | — |
| 9 | -226.23 | 3 | 358.76 | 2.61 | 0.046 | 0.7946 | — | -0.0960 | -0.0708 | — | — |
| 10 | -228.05 | 2 | 359.44 | 3.29 | 0.033 | 0.8300 | — | — | — | + | — |
| 11 | -228.28 | 2 | 359.79 | 3.64 | 0.028 | 0.8672 | — | — | — | — | -0.0369 |
| 12 | -227.07 | 3 | 360.07 | 3.92 | 0.024 | 0.8063 | — | — | -0.0659 | + | — |
| 13 | -227.15 | 3 | 360.19 | 4.04 | 0.023 | 0.7905 | — | -0.0837 | — | + | — |
| 14 | -224.45 | 5 | 360.39 | 4.24 | 0.020 | 0.9424 | + | — | -0.0086 | + | — |
| 15 | -224.45 | 5 | 360.39 | 4.24 | 0.020 | 0.9577 | + | 0.0101 | — | + | — |
| 16 | -224.45 | 5 | 360.40 | 4.25 | 0.020 | 0.9503 | + | — | — | + | -0.0120 |
| 17 | -227.29 | 3 | 360.41 | 4.26 | 0.020 | 0.8227 | — | -0.0876 | — | — | -0.0464 |
| 18 | -227.33 | 3 | 360.47 | 4.32 | 0.020 | 0.8472 | — | — | -0.0648 | — | -0.0421 |
| 19 | -224.53 | 5 | 360.51 | 4.36 | 0.019 | 0.9803 | + | 0.0102 | — | — | -0.0113 |
| 20 | -225.96 | 4 | 360.51 | 4.36 | 0.019 | 0.7601 | — | -0.0937 | -0.0723 | + | — |
| 21 | -224.53 | 5 | 360.52 | 4.37 | 0.019 | 0.9681 | + | — | -0.0063 | — | -0.0146 |
| 22 | -224.53 | 5 | 360.52 | 4.37 | 0.019 | 0.9782 | + | 0.0099 | -0.0024 | — | — |
| 23 | -226.11 | 4 | 360.75 | 4.60 | 0.017 | 0.7952 | — | -0.0980 | -0.0720 | — | -0.0537 |
| 24 | -228.00 | 3 | 361.50 | 5.35 | 0.012 | 0.8318 | — | — | — | + | -0.0345 |

Abbreviations: AIC = Akaike’s information criterion; logLik = Likelihood; *k* = Number of parameters in the model; *w_i_* = Model weight; PC1 = The first PCA axis to describe environmental factors; T_max6_ = Mean maximal temperature of June; Year = Capture year; Sex-m = Male sex.

**Table S5**. Candidate models for predicting probability of tick infestation under the effects of probability of mite infestation (MP) and environmental factors (A total of 7 models’ accumulative Akaike weight ≤ 0.95).

| Model | logLik | *k* | AIC | ΔAIC | *w_i_* | Intercept | MP | PC1 | T_max6_ | MP ×  PC1 | MP × T_max6_ |
| --- | --- | --- | --- | --- | --- | --- | --- | --- | --- | --- | --- |
| 1 | -221.81 | 5 | 453.70 | 0.00 | 0.503 | -2.5634 | -0.1158 | -0.1990 | — | -0.8955 | — |
| 2 | -221.73 | 6 | 455.56 | 1.86 | 0.198 | -2.5587 | -0.1179 | -0.2129 | 0.1881 | -0.8969 | — |
| 3 | -221.32 | 7 | 456.78 | 3.07 | 0.108 | -2.5518 | -0.1361 | -0.2340 | 0.1209 | -0.8293 | 0.2100 |
| 4 | -227.06 | 2 | 458.15 | 4.44 | 0.055 | -2.5235 | — | — | — | — | — |
| 5 | -226.72 | 3 | 459.48 | 5.78 | 0.028 | -2.5688 | — | -0.4388 | — | — | — |
| 6 | -226.82 | 3 | 459.67 | 5.97 | 0.025 | -2.5707 | 0.2114 | — | — | — | — |
| 7 | -226.99 | 3 | 460.01 | 6.31 | 0.021 | -2.5193 | — | — | 0.1846 | — | — |

Abbreviations: AIC = Akaike’s information criterion; logLik = Likelihood; *k* = Number of parameters in the model; *w_i_* = Model weight; PC1 = The first PCA axis to describe environmental factors; MP = Mite infestation probability; T_max6_ = Mean maximal temperature of June.

**Table S6**. Candidate models for predicting tick load under the effects of mite load (ML) and environmental factors (A total of 2 models’ accumulative Akaike weight ≤ 0.95).

| Model | logLik | *k* | AIC | ΔAIC | *w_i_* | Intercept | ML | PC1 | T_max6_ | ML ×  PC1 | ML × T_max6_ |
| --- | --- | --- | --- | --- | --- | --- | --- | --- | --- | --- | --- |
| 1 | -317.71 | 6 | 647.76 | 0.00 | 0.624 | -0.5323 | -0.2322 | -0.0690 | — | -0.1650 | — |
| 2 | -317.51 | 7 | 649.48 | 1.71 | 0.265 | -0.5271 | -0.2341 | -0.0695 | 0.1441 | -0.1663 | — |

Abbreviations: AIC = Akaike’s information criterion; logLik = Likelihood; *k* = Number of parameters in the model; *w_i_* = Model weight; PC1 = The first PCA axis to describe environmental factors; ML = Mite load; T_max6_ = Mean maximal temperature of June.
